# Changes in repair pathways of radiation-induced DNA double-strand breaks at the midblastula transition in *Xenopus* embryo

**DOI:** 10.1093/jrr/rrae012

**Published:** 2024-04-20

**Authors:** Ryosuke Morozumi, Naoto Shimizu, Kouhei Tamura, Makoto Nakamura, Atsushi Suzuki, Hiroko Ishiniwa, Hiroshi Ide, Masataka Tsuda

**Affiliations:** Program of Biomedical Science, Graduate School of Integrated Sciences for Life, Hiroshima University, Higashi-Hiroshima, 739-8526, Japan; Amphibian Research Center, Graduate School of Integrated Sciences for Life, Hiroshima University, Higashi-Hiroshima, 739-8526, Japan; Program of Mathematical and Life Sciences, Graduate School of Integrated Sciences for Life, Hiroshima University, Higashi-Hiroshima, 739-8526, Japan; Program of Mathematical and Life Sciences, Graduate School of Integrated Sciences for Life, Hiroshima University, Higashi-Hiroshima, 739-8526, Japan; Amphibian Research Center, Graduate School of Integrated Sciences for Life, Hiroshima University, Higashi-Hiroshima, 739-8526, Japan; Department of Physiology, Cardiovascular Research Institute, University of California, San Francisco, San Francisco, CA 94158, USA; Eli and Edythe Broad Center for Regeneration Medicine and Stem Cell Research, University of California, San Francisco, San Francisco, CA 94158, USA; Amphibian Research Center, Graduate School of Integrated Sciences for Life, Hiroshima University, Higashi-Hiroshima, 739-8526, Japan; Institute of Environmental Radioactivity, Fukushima University, Fukushima, 960-1296, Japan; Program of Mathematical and Life Sciences, Graduate School of Integrated Sciences for Life, Hiroshima University, Higashi-Hiroshima, 739-8526, Japan; Program of Biomedical Science, Graduate School of Integrated Sciences for Life, Hiroshima University, Higashi-Hiroshima, 739-8526, Japan; Program of Mathematical and Life Sciences, Graduate School of Integrated Sciences for Life, Hiroshima University, Higashi-Hiroshima, 739-8526, Japan; Division of Genetics and Mutagenesis, National Institute of Health Sciences, Kanagawa, 210-9501, Japan

**Keywords:** Xenopus tropicalis, ionizing radiation, DNA double-strand breaks, homologous recombination, nonhomologous end joining, midblastula transition

## Abstract

Ionizing radiation (IR) causes DNA damage, particularly DNA double-strand breaks (DSBs), which have significant implications for genome stability. The major pathways of repairing DSBs are homologous recombination (HR) and nonhomologous end joining (NHEJ). However, the repair mechanism of IR-induced DSBs in embryos is not well understood, despite extensive research in somatic cells. The externally developing aquatic organism, *Xenopus tropicalis*, serves as a valuable model for studying embryo development. A significant increase in zygotic transcription occurs at the midblastula transition (MBT), resulting in a longer cell cycle and asynchronous cell divisions. This study examines the impact of X-ray irradiation on *Xenopus* embryos before and after the MBT. The findings reveal a heightened X-ray sensitivity in embryos prior to the MBT, indicating a distinct shift in the DNA repair pathway during embryo development. Importantly, we show a transition in the dominant DSB repair pathway from NHEJ to HR before and after the MBT. These results suggest that the MBT plays a crucial role in altering DSB repair mechanisms, thereby influencing the IR sensitivity of developing embryos.

## INTRODUCTION

Ionizing radiation (IR) can cause a variety of DNA damages, altering its structure. The ability to repair DNA is crucial for recovery from DNA damage, preventing cell death and reducing the accumulation of mutations [1]. DNA double-strand breaks (DSBs), a type of DNA damage caused by IR, are the most biologically harmful type of DNA lesions and can lead to cell death [2]. DSBs can be repaired through two primary pathways: homologous recombination (HR) and nonhomologous end joining (NHEJ) [3]. These repair pathways are highly conserved among vertebrates, highlighting their importance in maintaining genome integrity [4]. Although the effects of IR are primarily studied in somatic cells, research on its effects on embryos is limited.

*Xenopus tropicalis*, an aquatic organism that develops externally, allows for detailed observation of anlagen morphogenesis. Despite its longer reproductive cycle compared to other model organisms, *Xenopus*’s abundant embryonic cells and ease of manipulation during early embryogenesis offer significant research potential. *Xenopus* enables biophysical and physiological approaches to understand developmental signals that can be extrapolated to higher vertebrates, including humans [5, 6]. It also serves as a bridge between conventional *in vitro* and preclinical mammalian assays in biomedical research and drug development [7–9]. Furthermore, *Xenopus* provides a valuable bioindicator for assessing radioactive contamination due to its heightened radiosensitivity when exposed to such stimuli [10], enabling rapid data collection. In the course of *Xenopus* development, a pivotal event occurs known as the midblastula transition (MBT). At this stage, the cell cycle undergoes a deceleration, leading to an extension of the G1 phase. Concurrently, the zygotic genome becomes transcriptionally active, instigating asynchronous cell division. In embryos, the pathways for apoptosis and DNA damage checkpoints differ before and after the MBT [11, 12]. The administration of hydroxyurea to embryos before the MBT results in apoptosis during gastrulation [13], while irradiation after the MBT induces cell cycle arrest [14]. However, there is a scarcity of studies directly comparing the repair mechanisms of DSBs between embryos subjected to irradiation before and after the MBT.

In this study, we examined the effects of X-ray irradiation on *Xenopus* embryos before and after the MBT. Our findings revealed that embryos before the MBT are more sensitive to X-rays compared to those after the MBT. We also compared the use of HR and NHEJ in repairing X-ray-induced DSBs before and after the MBT. Interestingly, we observed that embryos before the MBT showed greater sensitivity to X-rays when an NHEJ inhibitor was present, compared to when an HR inhibitor was present. In contrast, embryos after the MBT exhibited greater sensitivity to X-rays in the presence of an HR inhibitor than in the presence of an NHEJ inhibitor. These findings provide valuable insights into the unique DNA repair mechanism in *Xenopus* embryos that helps maintain genome integrity.

## MATERIALS AND METHODS

### Embryos

Embryos were prepared following previously described methods [15, 16]. In brief, *X. tropicalis* eggs were obtained through *in vitro* fertilization and embryos were cultured in 0.1 × Marc’s Modified Ringer’s (MMR) (100 mM NaCl, 2 mM KCl, 1 mM MgSO_4_, 2 mM CaCl_2_, 5 mM2-[4-(2-Hydroxyethyl)-1-piperazinyl]ethanesulfonic acid (HEPES) (pH 7.5)) with 50 μg/ml gentamycin on 1% agarose-coated dishes at 27°C. After fertilization, only embryos that were developing normally were selected for further study. Prior to irradiation, unfertilized eggs, uncleaved eggs and eggs that showed clear signs of delayed development were removed. Dead embryos were also removed, and MMR was replaced every 24 h. All experiments were conducted in accordance with the guidelines of the Animal Experimentation Ethics Committee of our institution and conformed to internationally accepted regulations.

### Irradiation

X-rays were produced using an OHMiC OM-303 M X-ray generator, operating at 70 kV and 3 mA, with a 0.2 mm Al filter. The distance from the source to the object was set at 300 mm. The dose rate and linear energy transfer of the X-rays were 1.46 Gy/min and 10 keV/μm, respectively. The Fricke dosimeter was used to measure the dose rate [17], while the estimation of LET was based on the published data [18]. The embryos were exposed to X-rays at 0.1× MMR at room temperature.

### Data availability

The datasets used in this study for the expression levels of DNA-PKcs and RAD51 are publicly accessible. They can be found on the National Center for Biotechnology Information (NCBI) Gene Expression Omnibus under the accession number GSE65785 [19].

### Treatments

For experiments involving DSB repair inhibitors, embryos were treated with 0.1× MMR containing either NU7026 (ab120970, Abcam) or RI-1 (ab144558, Abcam). This treatment started 1 h before irradiation and continued until 48 h postfertilization (hpf).

### Observation and analysis

At 48 hpf (late tailbud stage), tadpoles were anesthetized with 0.01% MS-222 (Sigma Chemical) and fixed in 5% formalin for a minimum of 1 h. Samples were observed under a microscope (SZ2-ILST; OLMPUS) at 5× magnification. The survival rate was calculated as follows: [Number of living embryos at 48 hpf]/[Number of living embryos before exposure to X-rays] × 100. The malformation rate was calculated as follows: [Number of embryos showing malformation at 48 hpf]/[Number of living embryos at 48 hpf] × 100.

## RESULTS

**Fig. 1:**
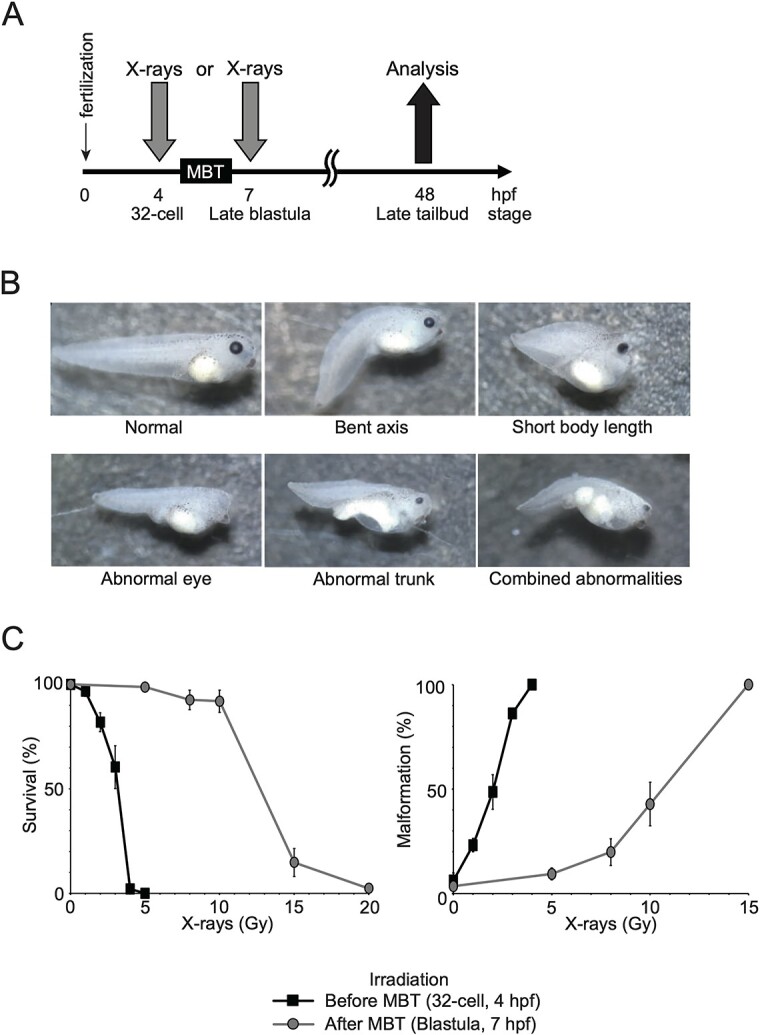
Effects of X-ray irradiation on the early development of *X. tropicalis* embryos. (**A**) Timeline of the experimental design: embryos were irradiated either before the MBT at 32 cell stage (4 hpf) or after the MBT at the late blastula stage (7 hpf). Survival and malformation were analyzed at the late tail bud stage (48 hpf). (**B**) Representative images: malformations observed at the late tail bud stage (48 hpf). (**C**) Survival and malformation rates: left: survival rates; right: malformation rates of *X. tropicalis* embryos irradiated with indicated doses of X-rays before the MBT (squares) and after the MBT (circles). The values are presented as mean and standard deviation from three independent irradiation experiments (50 embryos per replicate).

In this study, *Xenopus* embryos were exposed to X-ray irradiation either at 4 hpf (32-cell stage, pre-MBT) or at 7 hpf (late blastula stage, post-MBT). The survival and malformation rates were analyzed at 48 hpf (late tailbud stage) (Fig. 1A). Pre- and post-MBT irradiation resulted in multiple morphological malformations, bent axis, short body length, abnormal eye, abnormal trunk and combined abnormalities (Fig. 1B) (Supplementary Table 1). When embryos were exposed to 5 Gy of X-rays after the MBT stage, a 99% survival rate was observed (Fig. 1C, left graph). However, complete mortality occurred when embryos were exposed to 5 Gy of X-rays before the MBT stage. Irradiated embryos exhibited dose-dependent developmental malformations, with a higher rate of such abnormalities observed for irradiation before than after the MBT (Fig. 1C, right graph). These data suggest that embryos before the MBT are more sensitive to X-rays than those after the MBT in terms of survival and malformation.

**Fig. 2:**
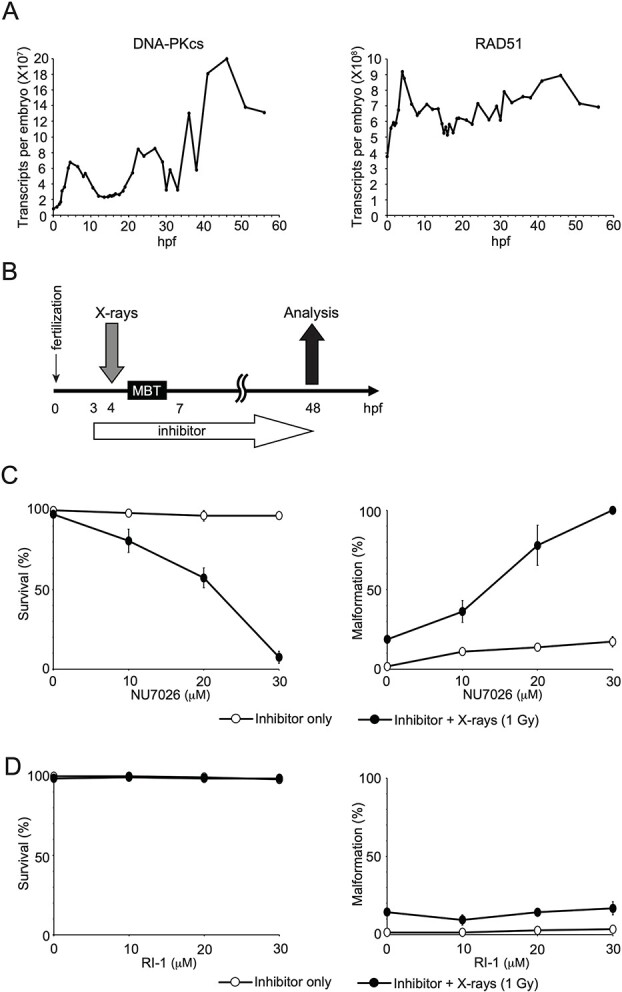
Effects of DNA DSB repair inhibitors on the survival and malformation of embryos irradiated before the MBT. (**A**) Time-courses of DNA-PKcs and RAD51 expression: time-courses of the expression levels of DNA-PKcs (left) and RAD51 (right) as revealed by polyA + RNA (RNA-seq) during early *Xenopus* development. Data sourced from Owens *et al.* [19]. (**B**) Experimental design timeline: embryos were irradiated before the MBT at 4 hpf, 1 h after the addition of inhibitors (NU7026 or RI-1) and incubated with inhibitors from 3 to 48 hpf. (**C**) NU7026 Treatment: left: survival rates; right: malformation rates of embryos treated with NU7026 (a DNA-PKcs inhibitor, open circles) or NU7026 + X-rays (1 Gy, closed circles). (**D**) RI-1 treatment: left: survival rates; right: malformation rates of embryos treated with RI-1 (a RAD51 inhibitor, open circles) or RI-1 + X-rays (1 Gy, closed circles).

In mitotic cells, the function of DSB repair pathways, HR and NHEJ, significantly influences sensitivity to IR [20]. However, it remains unclear whether DSB repair is linked to the IR sensitivity of embryos. To address this, we investigated the involvement of HR and NHEJ in the repair of X-ray-induced DSBs before and after the MBT. We employed DSB repair inhibitors and compared X-ray sensitivity in embryos before and after the MBT, in the presence or absence of the inhibitors. NHEJ is initiated by the Ku heterodimer, followed by the activation of DNA-dependent protein kinase catalytic subunit (DNA-PKcs) and the sealing of broken ends by various factors [21]. In somatic cells, the inhibition of DNA-PKcs significantly reduces the frequency of NHEJ [22]. On the other hand, RAD51 plays a critical role in HR, participating in the search for homologous sequences and strand pairing stages [23, 24]. In addition, DNA-PKcs and RAD51 are expressed during early *Xenopus* development (Fig. 2A) [19]. NU7026 and RI-1 were developed as inhibitors for DNA-PKcs and RAD51, respectively, and their activities were verified for human proteins. NU7026 acts as an ATP-competitive inhibitor of DNA-PKcs [25]. The DNA-PKcs protein sequence involved in ATP binding is highly conserved between human and *Xenopus* (Supplementary Fig. 1). Therefore, it is most likely that NU7026 also works as an ATP-competitive inhibitor of *Xenopus* DNA-PKcs, blocking NHEJ in *Xenopus*. RI-1 binds covalently to the surface of RAD51 protein at cysteine 319 and likely destabilizes an interface used to oligomerize RAD51 monomers into filaments on DNA [26]. The amino acid sequence of the RAD51 protein, including cysteine 319, is highly conserved in humans and *Xenopus* throughout its entire length (Supplementary Fig. 2). Thus, it is considered that RI-1 inhibit *Xenopus* RAD51, blocking HR in *Xenopus*.

Embryos were exposed to X-rays before the MBT (4 hpf) and treated with inhibitors from 3 to 48 hpf (Fig. 2B). It is important to note that without X-ray irradiation, concentrations of NU7026 and RI-1 ranging from 0 to 30 μM had little detectable effect on embryo development in terms of survival and malformation rates (Figs 2C and D). Therefore, we chose this concentration range for further analysis. When embryos were irradiated with 1 Gy before the MBT, there was a notable decrease in the survival rate and an increase in malformations in a dose-dependent manner with NU7026 (Fig. 2C) (Supplementary Table 2). However, this was not observed with RI-1 (Fig. 2D). These findings suggest that the inhibition of NHEJ by NU7026, but not HR by RI-1, increases the X-ray sensitivity of embryos before the MBT.

**Fig. 3:**
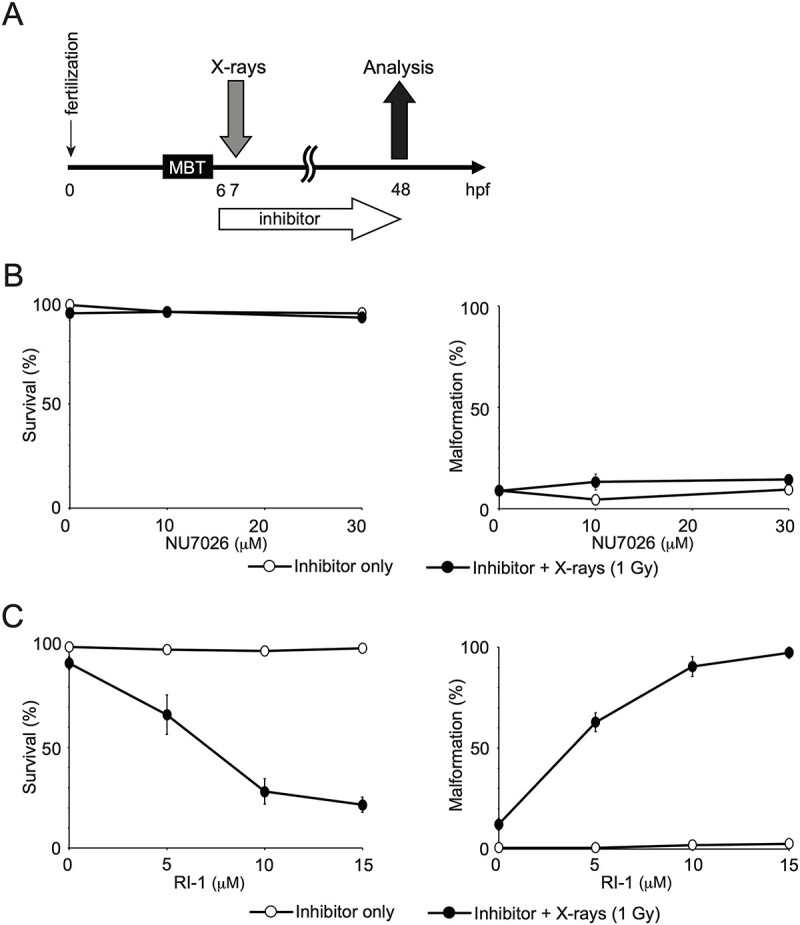
Effects of DSB repair inhibitors on the survival and malformation of embryos irradiated after the MBT. (**A**) Experimental design timeline: embryos were irradiated after the MBT at 7 hpf, 1 h after the addition of inhibitors (NU7026 or RI-1), and incubated with inhibitors from 6 to 48 hpf. (**B**) NU7026 treatment: left: survival rates; right: malformation rates of embryos treated with NU7026 (a DNA-PKcs inhibitor, open circles) or NU7026 + X-rays (1 Gy, closed circles). (**C**) RI-1 treatment: left: survival rates; right: malformation rates of embryos treated with RI-1 (a RAD51 inhibitor, open circles) or RI-1 + X-rays (1 Gy, closed circles).

We further analyzed the effect of NU7026 and RI-1 on the X-ray sensitivity of embryos after the MBT. Embryos were exposed to X-rays after the MBT (7 hpf) and treated with inhibitors from 6 to 48 hpf (Fig. 3A). The inhibitors (NU7026 and RI-1) alone had little effect on survival and malformation (Figs 3B and C). When embryos were irradiated with 1 Gy after the MBT, the rates of survival and malformation were comparable in the absence and presence of NU7026 (Fig. 3B). Conversely, with RI-1, the embryos irradiated after the MBT showed a decrease in the survival rate and an increase in the malformation rate in a dose-dependent manner of RI-1 (Fig. 3C) (Supplementary Table 3). These data suggest that inhibition of HR but not NHEJ increases the X-ray sensitivity of embryos after the MBT.

## DISCUSSION

**Fig. 4:**
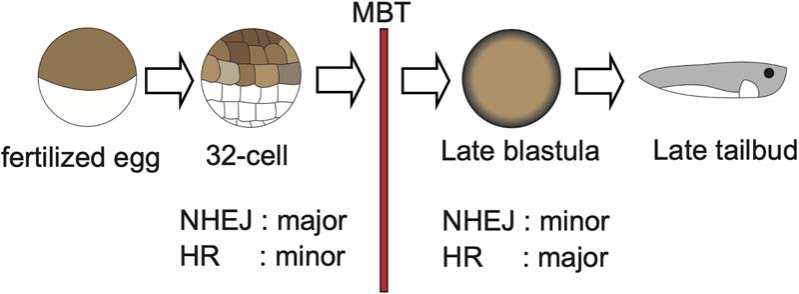
Model of changes in DSB repair pathways in developing *Xenopus* embryo. In developing *Xenopus* embryos, the major contributor to the repair of IR-induced DSBs is NHEJ before the MBT, whereas it changes from NHEJ to HR after the MBT.

Here we provide the evidence that NHEJ primarily contributes to the repair of X-ray-induced DSBs before the MBT in *Xenopus* embryos, while in embryos after the MBT, HR contributes more than NHEJ to the repair of DSBs (Fig. 4).

We observed enhanced IR sensitivity in *Xenopus* embryos before the MBT compared to later developmental stages (Fig. 1C). This is consistent with previous studies on zebrafish embryos, which also show the highest sensitivity to IR during early development [27–29]. Therefore, the difference in IR sensitivity before and after the MBT appears to be a conserved phenomenon between *Xenopus* and zebrafish. Several possibilities could explain the apparent difference in X-ray sensitivity before and after the MBT. First, the embryo before the MBT might lack sufficient amounts or types of proteins required for repairing IR-induced DNA damage, such as DSBs. Second, the cell division cycle before the MBT is notably shorter than that after the MBT, due to the absence of G1 and G2 phases. NHEJ is a rapid process, whereas HR is comparatively slower and requires more time for completion [30]. Consequently, in pre-MBT embryos, NHEJ is well-suited for efficiently repairing DSBs, while HR functions are less effective. This inadequacy may contribute to a heightened sensitivity to IR in pre-MBT embryos. Third, the prevalence of NHEJ and the relative deficiency of HR may lead to a decrease in the cell survival rate of embryos before the MBT. This proposition finds support in the observation that NHEJ predominates over HR in embryos before the MBT (Fig. 2). Additionally, the toxic influence of NHEJ-dependent DSB repair, in the absence of HR, results in abnormal joining of chromatid breaks, leading to the formation of a radial chromosome structure [31]. Nevertheless, further studies are imperative to elucidate and assess the DNA repair capabilities of early *Xenopus* embryos.

The expression level of DNA-PKcs is considerably lower than that of RAD51 before the MBT (Fig. 2A). However, according to the survival of irradiated embryos, NHEJ predominantly participates in the repair of X-ray-induced DSBs before the MBT in *Xenopus* embryos, whereas HR becomes more crucial than NHEJ in DSB repair after the MBT (Fig. 4). There are a few possible reasons for these discrepancies. First, the expression levels (the number of transcripts) of DNA-PKcs and RAD51 do not necessarily reflect their protein levels. Second, the expression and/or protein levels of HR-performing factors, other than RAD51, may be low in early development, making HR inoperative. These aspects should be fully clarified in future research.

## Supplementary Material

Supplementary_Figure_1_rrae012

Supplementary_Figure_2_rrae012

Supplementary_Table_1_rrae012

Supplementary_Table_2_rrae012

Supplementary_Table_3_rrae012
