# Supplementary figures and images for: Changes in repair pathways of radiation-induced DNA double-strand breaks at the midblastula transition in Xenopus embryo
Source: J Radiat Res. 2024 Apr 20;65(3):315–22. doi: 10.1093/jrr/rrae012 (PMC11115444; doi:10.1093/jrr/rrae012)

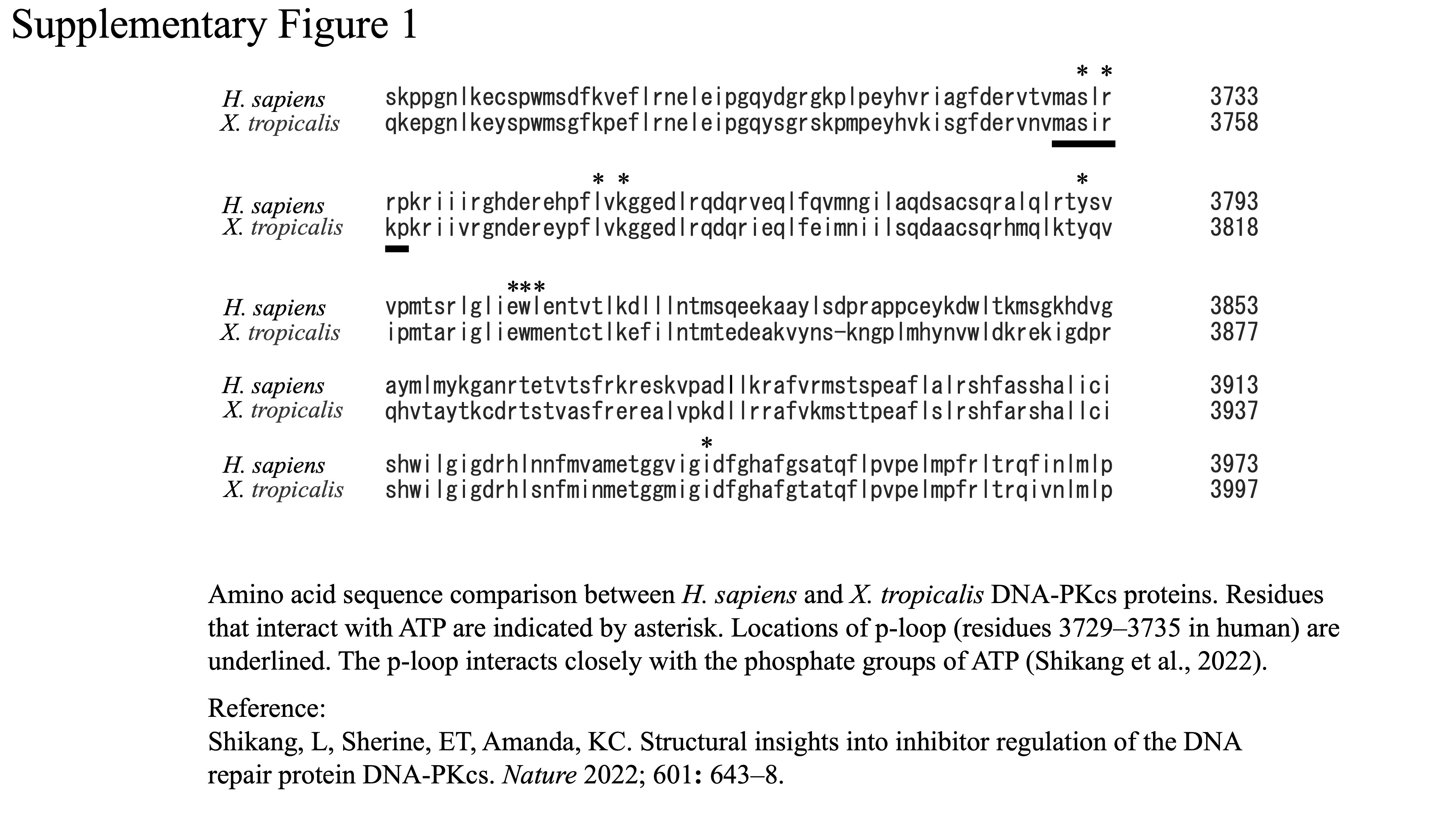

Supplement: Supplementary_Figure_1_rrae012 [file supplementary_figure_1_rrae012.jpeg]

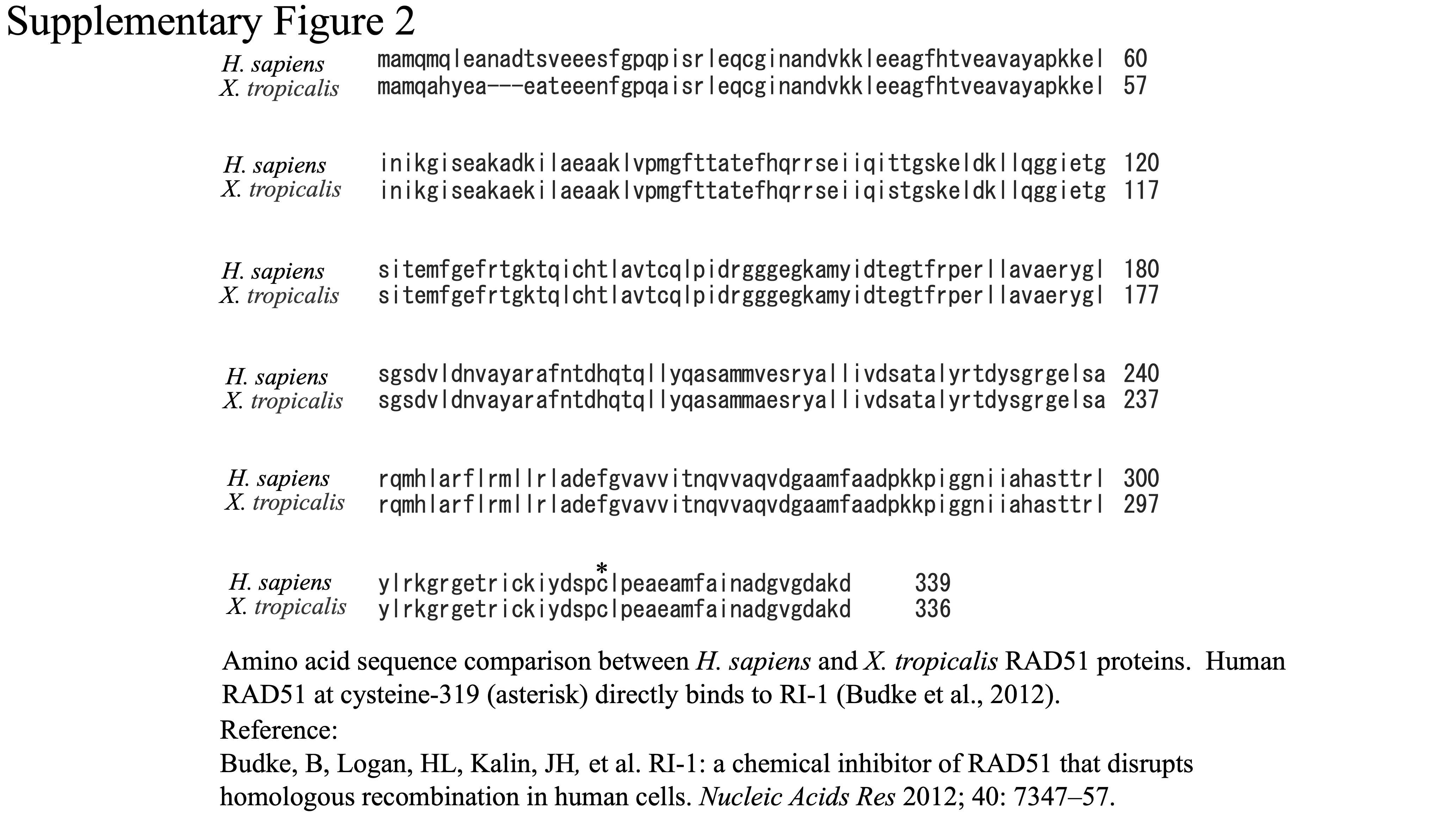

Supplement: Supplementary_Figure_2_rrae012 [file supplementary_figure_2_rrae012.jpeg]

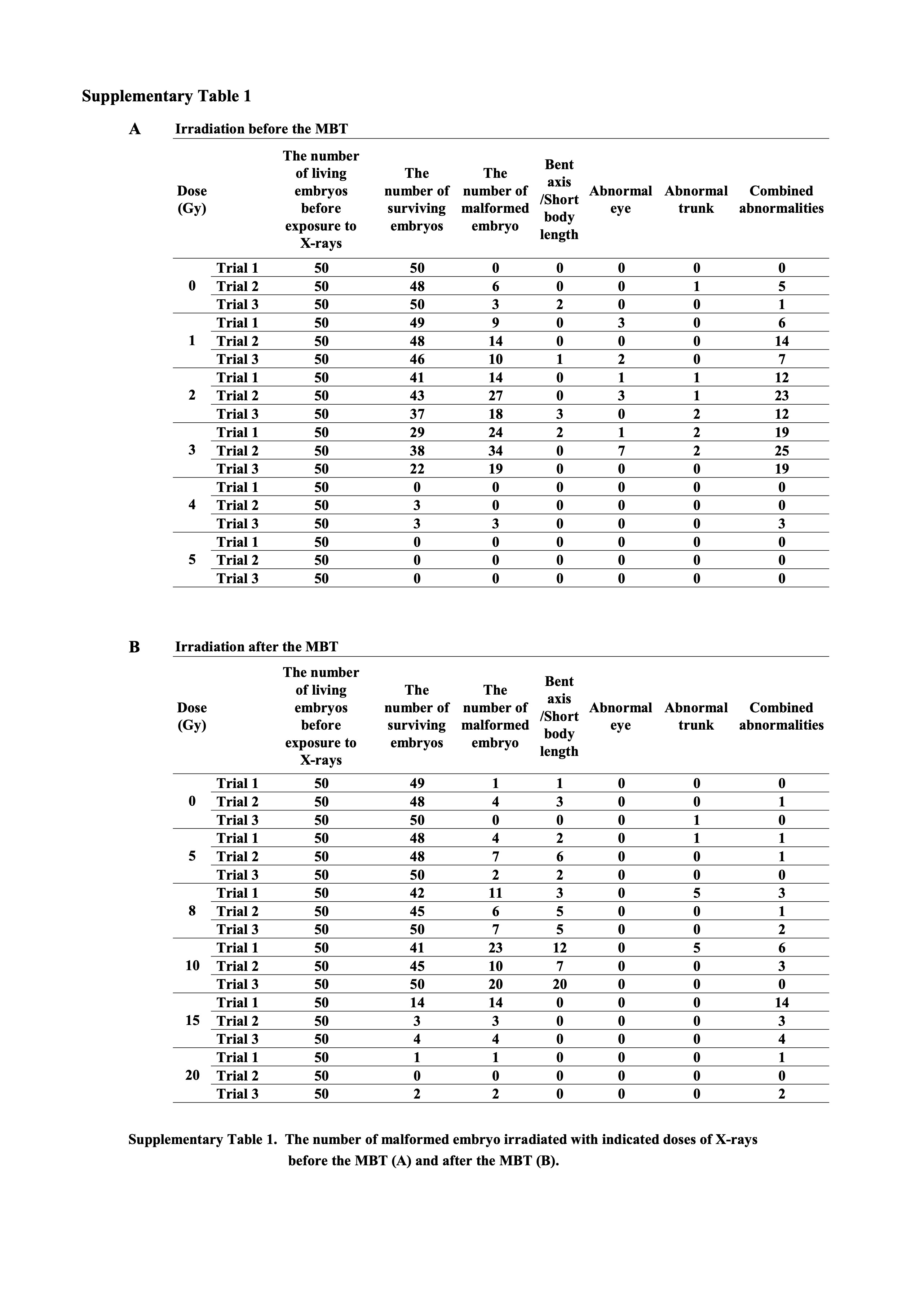

Supplement: Supplementary_Table_1_rrae012 [file supplementary_table_1_rrae012.jpeg]

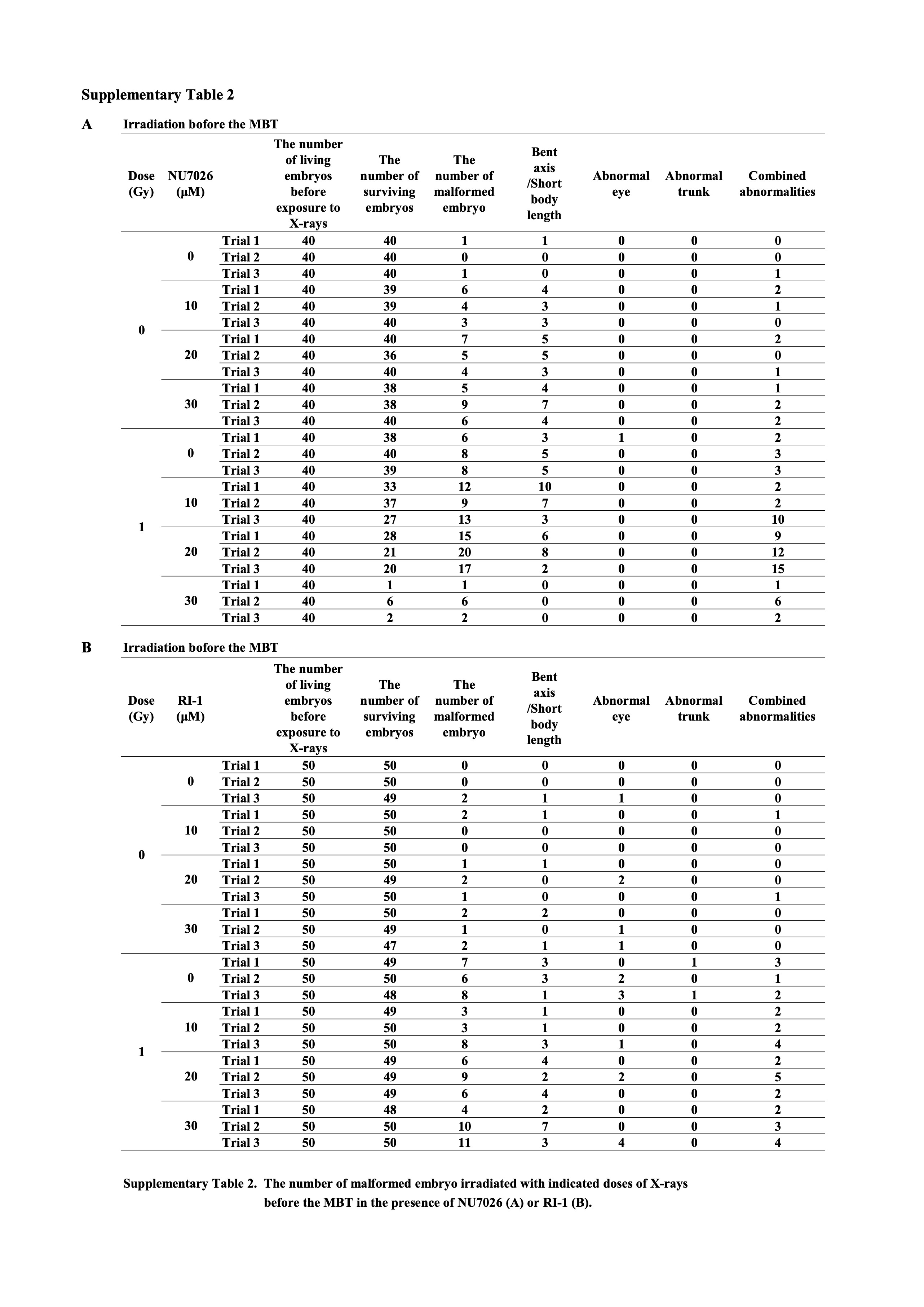

Supplement: Supplementary_Table_2_rrae012 [file supplementary_table_2_rrae012.jpeg]

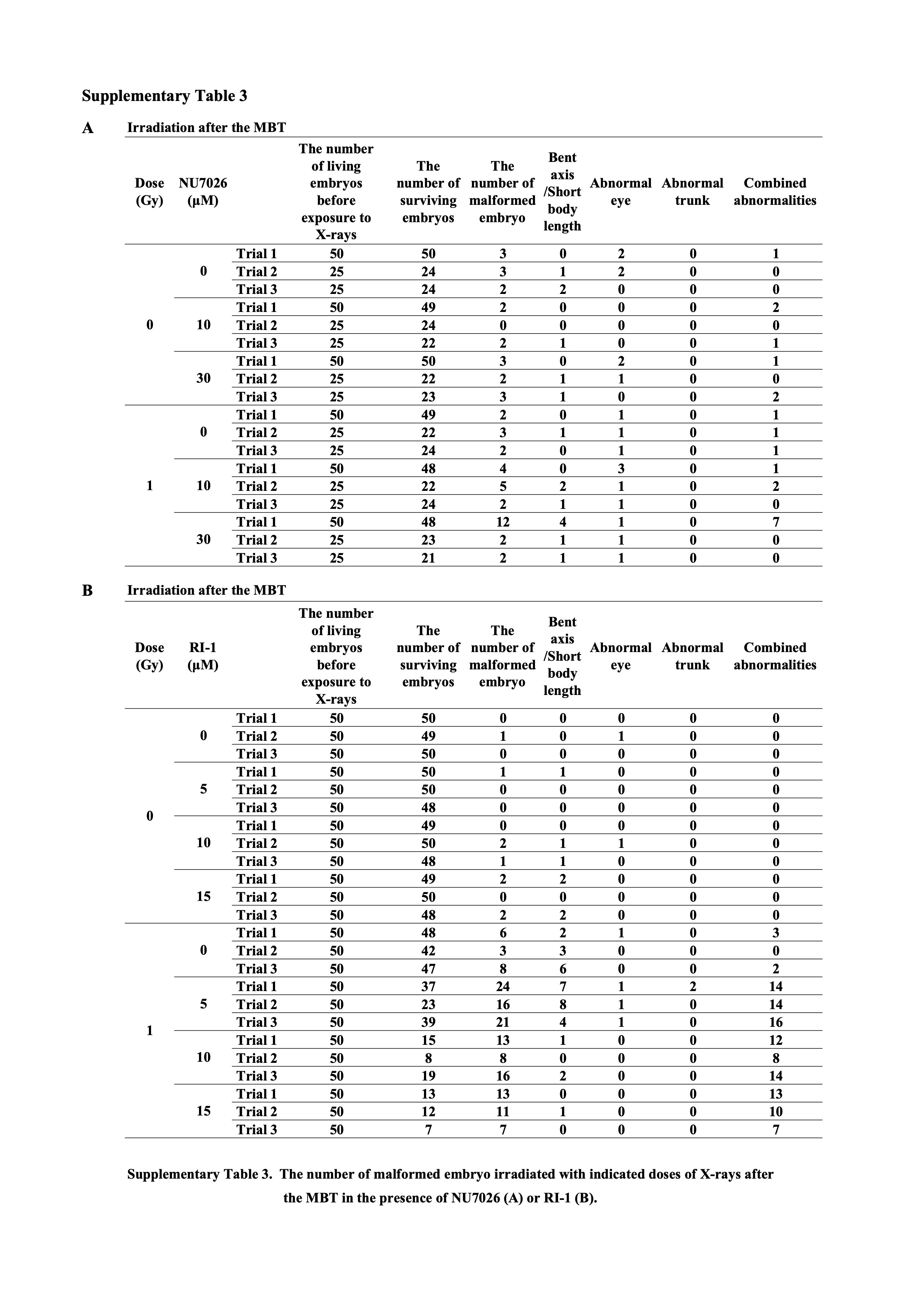

Supplement: Supplementary_Table_3_rrae012 [file supplementary_table_3_rrae012.jpeg]
